# How Consistent are Cognitive Impairments in Patients with Cerebellar Disorders?

**DOI:** 10.3233/BEN-2010-0271

**Published:** 2010-08-16

**Authors:** Dagmar Timmann, Irene Daum

**Affiliations:** ^1^Department of NeurologyUniversity of Duisburg-EssenEssenGermany; ^2^Institute of Cognitive NeuroscienceDepartment of NeuropsychologyRuhr-University BochumGermany

**Keywords:** IQ, intelligence, attention, verbal working memory, cognitive development

## Abstract

Many human lesion und functional brain imaging studies suggest involvement of the cerebellum in cognitive functions. However, negative and inconsistent findings are rarely discussed. It is still an open question as to which areas of cognition the cerebellum contributes, as well as how, and to what extent. Frequently cited earlier findings in one area of cognition have been challenged in more recent studies, that is the cerebellum may not be directly involved in attention. Furthermore, disorders in patients with acquired cerebellar disease are frequently mild and less severe compared to lesions of the corresponding areas of the cerebral cortex. Patients with cerebellar disease often perform within the normal range of neuropsychological test norms. This pattern is illustrated based on general intelligence and verbal working memory, which have been assessed by a large number of authors using comparable tests. Findings, however, appear to be more pronounced in individual cases with acute onset cerebellar disorders and in children, in particular with congenital disease. The review suggests that the inconsistencies in cognitive impairments may offer clues as to the nature of cerebellar cognitive involvement.

## 1. Introduction

The cerebellum is thought to support cognitive functions of the cerebral cortex, in particular of the frontal and parietal lobes [24]. There are many papers and reviews in the field, which provide good evidence that the cerebellum contributes to cognitive tasks, including executive and language functions ([8,40,84,92] for recent reviews). Negative and inconsistent findings, however, are frequently not discussed. The latter are the focus of the present review. This is to allow a more balanced discussion, but not to contradict that the cerebellum contributes to cognition. Findings in cerebellar patients are frequently mixed with respect to standard neuropsychological tests and negative findings are not uncommon. In order to relate observed abnormalities in human lesion studies to cerebellar dysfunction it is important that accompanying lesions of other brain areas are excluded. Extracerebellar lesions are common in focal cerebellar disorders, such as stroke, and cerebellar degeneration [93]. In a number of well-controlled human cerebellar lesion studies, clinical abnormalities appear to be mild and significantly less pronounced compared to lesions of the corresponding cerebral areas ( [21,22, 25,27] for review, [33,50,64,72,88,100,101]). However, other studies show more marked cognitive abnormalities [17,53,54,66,70,76]. Most notably Sherman and Schmahmann [70] report significant abnormalities in their original description of the 'cerebellar cognitive affective syndrome'. Because general intelligence and verbal working memory have been assessed by a large number of authors using comparable tests, results in these two areas are reviewed in more detail. Firstly, however, more recent findings in attention are discussed which show that, contrary to earlier assumptions, the cerebellum may not directly be involved in this area of cognition.

ISSN 0953-4180/10/$27.50 © 2010 – IOS Press and the authors. All rights reserved

## 2. Attention

There are specific areas of cognitive function with good evidence of involvement of the cerebellum. Verbal working memory and certain language functions may be the best examples. However, on the other hand, there is one area of cognition were earlier findings have recently been challenged. There is increasing evidence that the cerebellum may not be directly involved in attention.

Attention depends on cortical and subcortical structures, including prefrontal cortex, parietal cortex, thalamus, basal ganglia and midbrain [42,88]. The idea that the cerebellum may be involved in attention, was initially put forward by Courchesne and coworkers [20]. This is a reasonable assumption given that the cerebellum has been shown to have reciprocal anatomical connection with the prefrontal and parietal cortex [24]. Furthermore, the cerebellar posterior vermis is known to be important for saccadic control and it has been proposed that saccade programming and spatial visual attention might be based on the same neuronal substrates (premotor theory of attention [67]). Courchesne and coworkers extended their findings to a general theory of cerebellar involvement in cognition. The authors proposed that cognitive deficits observed in cerebellar patients might be secondary to impaired control of attention, a 'dysmetria of attention', analogous to the concepts of 'dysmetria of thought' and 'cognitive dysmetria' initially put forward by Jeremy Schmahmann [71] and Nancy Andreasen [7] respectively. Courchesne and coworkers initial findings of impaired spatial and non-spatial forms of attention in patients with cerebellar disease, however, have not been replicated or were related to motor demands of the task in more recent studies.

Covert allocation of spatial attention is commonly estimated from the difference in visual acuity threshold determined in the presence or absence of a spatial cue. Townsend et al. [95] reported deficits in covert shifts of spatial attention in cerebellar patients. Subjects were required to discriminate the orientation of the letter E. Some of the trials involved a spatial cue. Normal control subjects were able to shift attention to the attention-directing cue within 100 ms after onset. Cerebellar patients, however, were unable to use the cue to improve visual acuity when the time between cue and the target letter E was short. They needed 800-1200 ms of cue-target delay to increase visual acuity as good as the normal subjects. Findings of Townsend et al. [95] suggested that orienting of attention is slowed in the cerebellar patients.

Results of Townsend et al. [94,95] on deficits in spatial attention have been challenged by Yamaguchi et al. [102], Dimitrov et al. [22], Helmuth et al. [37] and Golla et al. [32]. None of the studies found deficits in visual spatial attention in cerebellar patients. In contrast to all previous studies, Golla and coworkers [32] assessed eye movements carefully. In one part of the study, subjects were required to perform visually guided saccades, that is overt shifts of spatial attention were tested. In the other part, covert shifts of attention were evoked by the need to discriminate the orientation of a Landolt C ring. Subjects had to fixate a central fixation point and visual acuity was assessed in the periphery. Landolt C rings were shown in two directions either with or without a preceding visual cue. Pressing a response button in the right hand indicated a gap at the top, and pressing the left button indicated a gap at the bottom. Importantly, eye movement recording ensured that the fixation point was fixated without the occurrence of abnormal eye movements, for example nystagmus. As expected, cerebellar patients presented with dysmetric saccades. In contrast, improvement in visual acuity induced by spatial cueing did not differ between cerebellar patients and controls (Fig. 1A). Thus, cerebellar patients were unimpaired in their ability to shift attention covertly, despite the presence of saccadic dysmetria. A subsequent cerebellar lesion study in the monkey performed by the same group showed exactly the same results [39]. Monkeys with lesions of the posterior vermis were able to use spatial attention to improve visual acuity, but suffered from disturbances of visually guided saccades. According to these findings, the posterior vermis appears important for the control of saccades only (that is overt shifts of attention), rather than being part of a common network for both overt and covert spatial shifts of attention.

The paradigms used by Townsend et al. [95] and Golla et al. [32] are very similar. Possible reasons for differences in findings are differences in motor demands and lack control of abnormal eye movements in the Townsend study. First, in the Townsend study subjects had to use a joystick to indicate four possible target directions. The latter is a more complex motor task. Ivry and coworkers have put forward the idea that deficits in attention are only present when subjects need to make more complicated movements. Second, the spatial cues have to be at the same point on the retina as the targets (E or Landolt C rings) in order to be effective. If involuntary eye movements such as nys-

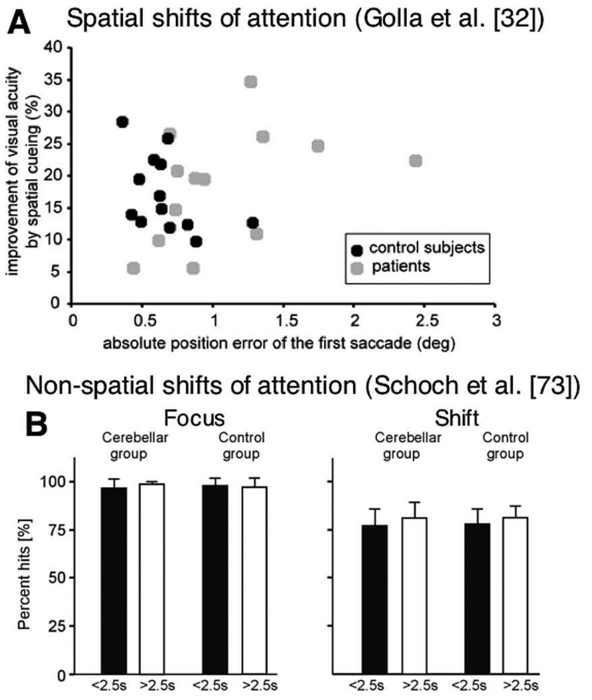

Fig. 1. (**A**) Spatial shifts of attention: The correlation between covert (y-axis) and overt (x-axis) shifts of spatial attention is shown in individual cerebellar patients ($n = 13$, grey dots) and control subjects ($n = 13$, black dots). Covert shifts of attention are quantified as improvement of visual acuity by valid spatial cueing (for details see text). Overt shifts of attention are measured as the absolute position error of the first saccade. Note that, despite saccade dysmetria (i.e. impaired overt shifts of attention), cerebellar patients improve visual acuity to the same extent as controls (i.e. unimpaired covert shifts of attention). (from [32] with permission). (**B**) Non-spatial shifts of attention: Mean percentage of correct target detection (PHITS) and standard deviation in the cerebellar ($n = 10$) and control ($n = 10$) group in a focus (left) and shift (right) attention task for trials of short time intervals $< 2.5$ s and long time intervals $> 2.5$ s. Note that children with cerebellar damage showed no significant impairment in rapid ($< 2.5$ s) shifts of attention. For details of experimental procedure see text. (from [73] with permission).

tagmus were present, and position in retina was shifted, this could have affected attentional improvements. Eye movements were not recorded in the Townsend study and therefore disorders in fixation may have gone undetected.

Very much in accordance with the initial findings regarding covert shifts of spatial attention, early findings of disordered non-spatial shifts of attention [4,5, 19] have been challenged in more recent studies ([36] for review, [72]). Akshoomoff and Courchesne [4] investigated non-spatial shifts of attention in a group of five children with surgical lesions and a young adult with a degenerative disorder. Subjects had to perform two tasks. In a focus attention task, subjects had to respond to a single rare stimulus, a red square or a high-pitched tone. In the shift attention task, subjects had to switch between the two rare stimuli, i.e. they had to respond in turn to the red square and the high-pitched tone. Subjects with cerebellar lesions were unimpaired in their ability to focus on the single rare stimulus, but showed a significant impairment in the ability to shift attention between stimuli of two modalities particularly within short interstimulus intervals ($< 2.5$ s). In accordance with these findings, Gottwald et al. [34,35] reported impaired performance in a divided attention task, but not in selective attention task in a group of adult patients with focal lesions.

A study of our group failed to replicate Akshoomoff and Courchesne's [4] initial findings in a larger group of ten children with acute focal cerebellar lesions [72]. A task was used which was closely related to the study of Akshoomoff and Courchesne. The ability of target detection did not significantly differ in the children with cerebellar lesions compared to the control children in both the focus and the shift attention task. In particular, children with cerebellar damage showed no significant impairment in rapid ($< 2.5$ s) shifts of attention (Fig. 1B).

The one other human lesion study using a similar paradigm is that by Ravizza and Ivry [60] in adult individuals with cerebellar diseases. Findings of their main statistical analysis were similar to our results, i.e. they did not find a significant group effect in rapid shifts of attention. The authors proposed a ceiling effect because of the higher level of performance in their patient group compared to the one of Courchesne and coworkers. Posthoc analysis of cerebellar patients alone revealed a difference in the short time interval comparing the shift and the focus attention task, which approached significance.

In a subsequent experiment reported in the same paper, Ivry and coworkers reduced the motor requirements of the shifting attention task. They compared performance in a second group of adult patients with cerebellar damage in the original shift task where subjects had to press the response key to each rare stimulus with a shift task where they had to overtly respond to targets in one modality only. The patients showed significantly better performance in the task with lower motor requirements in the short time interval. Ivry and coworkers concluded that deficits seen in the first experiment are likely due to motor performance deficits in cerebellar patients.

In both Akshoomoff and Courchesne's [4] and our study [72], subjects had to respond to each of the rare stimuli by pressing a response button. Cerebellar patients assessed by Akshoomoff and Courchesne showed more marked motor impairment compared to patients included in our study. Lack of significant motor impairment may explain why no deficits in shifting attention were observed in our group of children with cerebellar lesions. Negative findings have also been reported in other attentional tasks in cerebellar lesion studies [37]. Helmuth et al. [37] failed to observe deficits in adult patients with cerebellar disorders in a series of non-spatial (and spatial) attention shifting tasks. Again, the authors suggested that differences in findings were due to more demanding motor requirements in Courchesne's studies. Likewise, abnormalities in other standard neuropsychological tests of attention observed in cerebellar patients (trail-making test: [26,34,35,51]; but see [38] for a negative result; digit symbol test [38,51,70]) have been related to the significant oculomotor, motor and/or working memory demands of the tasks [36].

The view that disorders in attention are related to motor requirements of the task is further supported by functional MRI (fMRI) studies. Results of Ivry's group show that switching attention between dimensions without a motor response does not produce stronger cerebellar activation compared with the focus attention condition [13]. The authors proposed that the cerebellum is involved in response reassignment but not switching attention. Likewise, Corbetta et al. [18] failed to observe cerebellar BOLD responses in association with covert shifts of spatial attention.

Findings challenge conclusions based on previous fMRI studies in favor of cerebellar involvement in attention [6,47]. Although Allen et al. [6] showed that different areas were activated in the cerebellum during a pure motor control task (hot spot in the anterior cerebellum) and a pure attention task (hot spot in the posterior cerebellar hemisphere), cerebellar activation may still be attributed to motor performance in the attention task. In the pure attention task, subjects did not have to press a response button but had to count the rare stimuli. Inner speech is known to activate the posterior cerebellar hemisphere [3]. Furthermore, Allen et al. [6] used a focus but not a shift attention paradigm. Based on Courchesne's initial findings of unimpaired focus attention in cerebellar children, attention related cerebellar activation during the focus task appears unexpected. In the fMRI study by Le et al. [47], the contrast of a shifting attention minus a focus attention condition revealed significant activation in the lateral cerebellar hemisphere. However, in contrast to Ivry's study, a covert shifting attention condition was not included.

Thus, possible involvement of the cerebellum in attentional tasks is less clear than frequently assumed. The cerebellum does not appear to be directly involved in attentional processes. At least parts of the deficits in attention following cerebellar lesions appear to be attributable to motor requirements of the tasks.

## 3. General intelligence

Many patients with circumscribed lesions of the cerebellum, which have been acquired after birth, perform within the normal range on IQ tests. Although various abnormalities are reported in specific tests, many studies report no significant differences between mean IQ measures in cerebellar and normal control groups (Table 1). This holds for children and adults with chronic lesions of various etiologies, in particular cerebellar tumor removal in children and adolescents and following stroke and degenerative disorders in adults. However, as described below, findings in patients with acute onset cerebellar lesions are more mixed. Furthermore, markedly reduced IQ has been reported in children with congenital cerebellar malformations and very preterm children with cerebellar lesions.

Our own group assessed general IQ in adults and children with chronic and acute cerebellar lesions. As a reliable measure of intelligence, the standard progressive matrices test (SPM) without time restrictions was used in adults, and the coloured progressive matrices test (CPM) in children [59]. The CPM is a short version of the standard progressive matrices test (SPM) and recommended for children. Patterns of increasing complexity are shown with one segment missing. Subjects have to indicate the missing segment out of four possibilities. The absence of time restrictions minimizes the potential influence of the patient's motor performance deficits on the IQ.

We tested adult patients with chronic, pure cerebellar degeneration [23,56,63,89,91]. In each study SPM score percentiles were within the normal range in the cerebellar patients and there was no statistically significant difference compared to age, sex and education matched controls. The major aim of these studies was to assess associative learning in cerebellar patients and IQ was used as a matching variable. None of the recruited control subjects, however, were excluded based on their IQ measures. Therefore, group comparison between patients and normal subjects appears justified. In our studies group size was comparatively small ($n =$ 8–12) and patients with different disorders, which led to pure cerebellar cortical degeneration (that is, spinocerebellar ataxia type 6, SCA6; sporadic adult onset ataxia, SAOA; and autosomal dominant ataxia type III, ADCAIII), were included. Results are, however, consistent with a more recent study in a larger and more homogenous group of subjects. Suenaga et al. [85] used Raven's coloured progressive matrices (CPM) in 18 SCA6 patients and 21 controls. Again, IQ measures were within the normal range and not significantly different compared to a control group. Leggio et al. [48] applied CPM in a group of chronic cerebellar patients, 11 with focal lesions of the right cerebellum, 9 with focal lesions of the left cerebellum and 14 with cerebellar degeneration. All cerebellar subjects performed in the normal range. In the patients with degenerative disorders, but not the focal cerebellar patients, group mean IQ measures were below mean IQ measures in the controls. The degenerative group, however, included various etiologies and not all of them represented pure cerebellar disease. Finally, our group performed the CPM in a study in children and adolescents with acute surgical lesions of the cerebellum. CPM scores were within the normal range and not significantly different compared to age, sex and school education matched controls [72]. The same negative findings were reported in children with removal of benign cerebellar tumors tested in the chronic stage [1,97].

Scores in the normal range have also been reported in early studies when given either the complete Wechsler Adult Intelligence Scale (WAIS) or subtests of this instrument both in patients with degenerative and focal cerebellar lesions [21,22]. The WAIS is used to assess global cognitive abilities in terms of verbal and non-verbal intelligence [98]. Globas et al. [31] used a short German version of the WAIS in twelve patients with SCA6 and twelve healthy controls. No significant group difference was observed. In these three studies, however, IQ was used as a matching variable and group differences between patients and controls may have been missed.

This was not the case in the studies by Garrad et al. [28] and Leggio et al. [48]. Garrad et al. found no decline in IQ in a group of 9 patients with SCA6. One SCA6 patient performed below the normal range. Leggio et al. investigated IQ in a large number of chronic cerebellar patients ($n = 60$) based on WAIS. There was no significant difference between the group of all cerebellar patients and the control group. Patients were subdivided in a group with focal (due to stroke or surgery) lesions of the right and left cerebellum and patients with cerebellar degeneration. In the latter group mean IQ was significantly lower than in the controls, although all patients performed within the normal range. There was partial overlap with the patients tested with the CPM by the same group [48] and not all degenerative patients presented with a pure cerebellar syndrome.

The WAIS or a subset of the WAIS has also been administered in adults with cerebellar stroke. In their seminal paper, Schmahmann and Sherman [70] report a decline in IQ. Findings were most prominent in patients with acute stroke (and acute onset cerebellar disease of other etiology) and tended to be transient. Exner et al. [26] tested stroke patients at least six months after the insult. No significant group difference was observed between patients with strokes within the territory of the posterior inferior cerebellar artery (PICA), patients with strokes within the territory of the superior cerebellar artery (SCA) and controls. Malm et al. [51] and Hokkanen et al. [38] assessed IQ in patients with acute cerebellar stroke. Hokkanen et al. reported no abnormalities of IQ compared to a control group at three months after the insult. Malm et al. did not report any changes in intelligence with patients being tested acutely, at 4 and at 12 months.

Another group of patients, in which IQ has frequently been assessed are children and adolescents who have been operated for benign cerebellar tumors, most of them pilocytic astrocytoma, who do not require adjuvant radiotherapy and surgery following tumor removal. One potential complication in these studies is that the presence of hydrocephalus observed in many tumor patients can contribute to cognitive impairment [1,69], emphasizing the need for researchers to evaluate this factor. In contrast to studies in adult subjects and our own studies in children using CPM, in these studies no control group is assessed but findings are compared to normative data given in the literature.

Riva and Giorgi [66] examined 26 patients with acute tumor surgery with neuropsychological testing being performed at least 5–6 weeks after surgery. Although age – appropriate Wechsler scale scores showed a decline, scores were within the normal range except for one child, who had developed an autistic behaviour. Observed changes were transient. Accordingly, preserved general intelligence has been reported in the majority of studies in children with chronic surgical lesions [2,68,69,81]. There are two exceptions. Kirschen et al. [43] reported IQ within or above the normal range in all but one of twelve children after benign cerebellar tumor resection. Mean IQ, however, was significantly below the controls. Significant differences of group means have also been reported in a very large group

Table 1

Summary of IQ measures reported in human cerebellar lesion studies

| Study | **Cerebellar condition** (n/age) | IQ test | IQ score | Group comparison |
|---|---|---|---|---|
| *Adults: cerebellar degeneration* | | | | |
| Daum et al. [21] | - pure cerebellar (degeneration and focal; n = 13; 53.4 SD 14.8 yrs.)<br>- cerebellar plus brainstem (n = 6; 58.7 SD 7.8 yrs.)<br>- controls (n =13; 52.6 SD 13.8 yrs.) | Wechsler, short version | FSIQ<br>- pure cerebellar: 114.5 SD 11.7<br>- cerebellar plus brainstem: 110.8 SD 13.2<br>- controls: 120.88 SD 7.7 | p = 0.121 (ANOVA) |
| Dimitrov et al. [22] | - SCA1, SCA3 (n = 10; 51 SD 16 yrs.) | Wechsler | FSIQ<br>- cerebellar: 102 SD 16 | |
| Drepper et al. [23] | - SAOA (n = 9; 63.89 SD 10.83 yrs.)<br>- controls (n = 10; 61.80 SD 8.09 yrs.) | SPM | SPM score (percentiles)<br>- cerebellar: 76.11 SD 27.25<br>- controls: 82.00 SD 12.06 | p = 0.543 (Mann-Whitney U) |
| Timmann et al. [89] | - SAOA, ADCAIII, SCA6 (n = 9; 56.67 SD 9.41 yrs.)<br>- controls (n = 9; 55.0 SD 8.46 yrs.) | SPM | SPM score (percentiles)<br>- cerebellar: 85.56 SD 11.3<br>- controls: 90.56 SD 7.68 | p = 0.289 (t test) |
| Maschke et al. [56]<br>Timmann et al. [91] | - SAOA, ADCAIII, SCA6, EA2 (n = 8; 52.2 SD 6.2 yrs.)<br>- controls (n = 8; 52.9 SD 7.9 yrs.) | SPM | SPM score (percentiles)<br>- cerebellar: 84.38 SD 17.2<br>- controls: 83.13 SD 22.51 | p = 0.706 (t test) |
| Richter et al. [63] | - SAOA, ADCAIII, SCA6, EA2 (n = 12; 50.3 SD 10.4 yrs.)<br>- controls (n =12; 53.3 SD 10.9 yrs.) | SPM | SPM score (percentiles)<br>- cerebellar: 85.4 SD 14.7<br>- controls: 91.7 SD 5.4 | p > 0.15 ( t test) |
| Globas et al. [31] | - SCA6 (n = 12; 57.5 SD 10.4 yrs.)<br>- controls (n = 12; 59.0 SD 5.6 yrs.) | Wechsler, short version | FSIQ<br>- cerebellar: 119.5 SD 10.1<br>- controls: 124.4 SD 8.4 | p = 0.22 (oneway-ANOVA) |
| Suenaga et al. [85] | - SCA6 (n = 18; 64.1 SD 11.9 yrs.)<br>- controls (n = 21; 63.0 SD 9.4 yrs.) | CPM | raw score (max. 36)<br>- cerebellar: 27.8 SD 5.0<br>- controls: 30.3 SD 3.6 | n.s. (Mann-Whitney U) |
| Garrard et al. [28] | - SCA6 (n = 9; 35-75 yrs.) | Wechsler | FSIQ<br>- SCA6: 111.44 SD 14.68 | |

Table 1, continued

| | | | | |
|---|---|---|---|---|
| *Adults: chronic focal lesions (mainly cerebellar stroke)* | | | | |
| Exner et al. [26] | - PICA (n = 6; 62 SD 5 yrs.)<br>- SCA (n = 5, 55 SD 3 yrs.)<br>- controls (n = 11; 57 SD 5 yrs.) | Wechsler, short version | FSIQ<br>- PICA: 99 SD 22<br>- SCA: 110 SD 13<br>- controls: 108 SD 16 | n.s. (Kruskal-Wallis) |
| Leggio et al. [48] | - right focal lesions (n = 21; 53.29 SD 18.44 yrs.)<br>- left focal lesions (n = 21; 49.76 SD 17.98 yrs.)<br>- cerebellar degeneration (n = 18; 42.88 SD 12.81)<br>- controls (n = 69; 43.78 SD 15.96 yrs.) | Wechsler | FSIQ<br>- right focal: 100.71 SD 14.56<br>- left focal: 97.57 SD 9.88<br>- degeneration: 90.17 SD 17.58<br>- controls: 106.17 SD 12.45 | controls vs. right focal vs. left focal vs. degeneration: p = 0.001 (oneway-ANOVA); controls vs. degeneration (p = 0.000; posthoc Bonferroni) |
| Leggio et al. [48] | - right focal lesions (n = 11; 48.18 SD 20.72 yrs.)<br>- left focal lesions (n = 9; 60.63 SD 7.42 yrs.)<br>- cerebellar degeneration (n = 14; 49.36 SD 11.08 yrs.)<br>- controls (n = 132; 47.02 SD 17.33 yrs.) | SPM, short version | Raw score<br>- right focal: 28.50 SD 3.52<br>- left focal: 29.60 SD 3.56<br>- degeneration: 24.74 SD 8.23<br>- controls: 29.50 SD 2.52 | controls vs. right focal vs. left focal vs. degeneration: p = 0.001 (oneway-ANOVA); controls vs. degeneration (p = 0.000; posthoc Bonferroni) |
| *Adults: acute focal lesions (mainly cerebellar stroke)* | | | | |
| Schmahmann and Sherman [70] | n = 20 (48.2 range 23-74 yrs.):<br>- acute stroke (n = 12)<br>- acute tumor removal (n =1)<br>- chronic stroke (n = 1)<br>- acute cerebellitis (n = 3)<br>- cerebellar degeneration (n = 3) | Wechsler | Z score<br>FSIQ –1.0 (SD 0.123)<br>VIQ –0.93 (SD 0.145)<br>PIQ –1.3 (SD 0.127) | compared to normative data:<br>FSIQ p = 0.0002<br>VIQ p < 0.0001<br>PIQ p = 0.0006 |
| Malm et al. [51] | - acute stroke (n = 24; 36.9 SD 6.5 yrs.)<br>- controls (n = 14; 33.1 SD 4.8 yrs.) | Wechsler | FSIQ/PIQ<br>- cerebellar: 101 SD 10.6 / 102.6 SD 10.7<br>- controls: 106.21 SD 17.9 / 107.57 SD 14.7 | n.s. (ANOVA) |

Table 1, continued

| | | | | |
|---|---|---|---|---|
| Hokkanen et al. [38] | - acute stroke (n = 26; 47 SD 11 yrs.)<br>- controls (n = 14; 46 SD 11 yrs.) | Wechsler, short version | Mean standard scores in subtests<br>- cerebellar: 12.1 SD 1.6<br>- controls: 12.7 SD 1.5 | n.s. ( t test) |
| *Children: acute focal lesions (surgery for cerebellar tumors)* | | | | |
| Riva and Giorgi [66] | - astrocytoma (n = 15 mean 10.2 yrs.)<br>- medulloblastoma (n = 11 mean 7.10 yrs.) | Wechsler | Z scores VIQ/PIQ<br>- right cerebellar: -1.4/+0.3<br>- left cerebellar: +0.2/-1.3<br>- vermis:<br>mutism, speech disturbance: +0.5/-0.1<br>mutism, language disturbance: +0.8/+0.4<br>- vermis:<br>minor behavioural disturbance: -0.6/-0.8<br>autistic behaviour: -1.8/-3.4 | Z scores > 2 were considered abnormal |
| Schoch et al. [72] | - acute tumor removal (n = 10; radiation and chemotherapy in 2/10; 13.1 SD 2.7 yrs.)<br>- controls (n = 10; 13.8 SD 2.8 yrs.) | CPM/SPM | CPM/SPM scores (percentiles)<br>- cerebellar: 56.8 SD 26<br>- controls: 69.9 SD 19.4 | p = 0.2 (unpaired t test) |
| Beebe et al. [9] | - astrocytoma (n = 103; tested 108 SD 78 days after surgery; 8.5 SD 4.1 yrs.) | Wechsler | FSIQ<br>- cerebellar (91/103 tested): 96 SD 14 | compared with normative data:<br>p = 0.003 (t test) |
| *Children: chronic focal lesions (surgery for benign cerebellar tumors)* | | | | |
| Steinlin et al. [81] | - benign cerebellar tumors (n = 24; mean 15.04 yrs.) | Wechsler | FSIQ<br>mean 99.1<br>(n = 3/24 < 85) | n.s. (–0.28 t test) |
| Aarsen et al. [1] | - astrocytoma (n = 23; mean 12 yrs. 9 months) | CPM | Z score 0.30 SD 1.00 | n.s. |
| Kirschen et al. [43] | - astrocytoma (n = 12; 12.5 SD 4.1 yrs.)<br>- controls (n = 12; age-matched) | Wechsler, short version | FSIQ<br>- cerebellar: 105.7 SD 13<br>- controls: 119.5 SD 15.9 | P = 0.029 ( t test) |
| Aarsen et al. [2] | - astroyctoma (n = 35; 29/35 | Wechsler | FSIQ | compared with normative |

Table 1, continued

| | | | | |
|---|---|---|---|---|
| | cerebellum, 6/35 brainstem) | | - cerebellar 97 SD 16 | data:<br>n.s. (chi-square test) |
| *Children: chronic focal lesions (surgery for benign vs. malign cerebellar tumors)* | | | | |
| Rønning et al. [69] | - astrocytoma (n = 12 ; 23.5 SD 3.1 yrs.)<br>- medulloblastoma (n =11; radio- and chemotherapy, 23.1 SD 4.9 yrs.) | Wechsler, short version | FSIQ<br>- astrocytoma: 94.0 SD 11.6<br>- medulloblastoma: 82.6 SD 18.1 | astrocytoma vs. medulloblastoma: p =0.11 (t test) |
| Vaquero et al. [97] | - medulloblastoma (n = 7, chemo- and radiotherapy; 13.59 SD 1.62 yrs.)<br>- astrocytoma (n = 13; 11.5 SD 3.3 yrs.)<br>- controls (n = 12; 11.33 SD 1.82) | SPM | IQ<br>- medulloblastoma: 85.85 SD 14.06<br>- astrocytoma: 93 SD 16.8<br>- controls: 101.58 SD 10.35 | - medulloblastoma: p = 0.017<br>- astrocytoma: n.s. (Mann-Whitney U) |
| Roncadin et al. [68] | - astrocytoma (n = 29; 17.43 SD 7.11 yrs.)<br>- medulloblastoma (n = 29; radio- and chemotheray; 23.54 SD 8.33 yrs.) | Wechsler | FSIQ<br>- astrocytoma: 98.52 SD 15.64<br>- medulloblastoma: 79.38 SD 12.02 | astrocytoma vs. medulloblastoma:<br>p < 0.001 (t test) |
| Puget et al. [58] | - malign cerebellar tumors (n = 61; radio-and chemotherapy; mean age at diagnosis 6.0 yrs., mean duration since diagnosis 4.7 yrs.) | Wechsler | FSIQ<br>- both dentate nuclei intact: 83<br>- both dentate nuclei damaged: 65 | dentate nuclei intact vs. damaged:<br>p = 0.009 |
| *Children: congenital non-progressive ataxia (cerebellar malformations)* | | | | |
| Steinlin et al. [80] | congenital non-progressive cerebellar ataxia (n = 11; 11.9 range 8-28 yrs.) | Wechsler , short version | FSIQ<br>- cerebellar: mean 62, range 30-90<br>- n = 10/11 < 85 (normal limits) | |
| Tavano et al. [86] | congenital non-progressive cerebellar ataxia (n = 27; 11.06, range 3.06-34 yrs.) | Wechsler | FSIQ<br>- n = 20/27 < 70<br>- n = 5/27 borderline IQ (70-85)<br>- n = 2/27 normal IQ (105, 91) | |
| Tavano et al. [86] | cerebellar agenesis (n = 1; 34 yrs.) | Wechsler | FSIQ = 69 | |
| Timmann et al. [90] | cerebellar agenesis (n = 1; 59 yrs.) | SPM | IQ = 87 | |
| Richter et al. [65] (same as in [90]) | cerebellar agenesis (n = 1; 61 yrs.) | Wechsler | FSIQ = 66 | |

ADCA III autosomal dominant cerebellar ataxia type III („pure cerebellar“); CPM coloured progressive matrices test; EA2 episodic ataxia type 2; FSIQ full scale intelligence quotient; PICA stroke within the territory of the posterior inferior cerebellar artery; PIQ performance intelligence quotient; SAOA sporadic adult onset ataxia; SCA stroke within the territory of the superior cerebellar artery; SCA1,3 spinocerebellar ataxia type 1,3 (extracerebellar involvement is common); SCA6 spinocerebellar ataxia type 6 („pure“ cerebellar); SD standard deviation; SPM standard progressive matrices test; VIP verbal intelligence quotient; Wechsler age- and language appropriate Wechsler intelligence scale.

of children. Beebe et al. [9] examined 103 children after resection of benign tumors (age range 3–18 years) within the first year after surgery. Intelligence was assessed with the Wechsler scale in 91 of the 103 patients. In the sample as a whole, mean scores were generally within the average normal range. However compared to the normative mean in the true population for full scale IQ, performance IQ, and to a lesser degree verbal IQ mean values were significantly reduced.

The picture is different when children and adolescents with malign cerebellar tumors are assessed. In these groups, IQ measures are significantly below the normal range [68,69,97]. In a recent study Puget et al. [58] tested 61 children with chronic malignant posterior fossa tumors, tested at least six months after adjuvant treatment has been finished. Mean full-scale IQ was 83 in a subgroup of children in which both dentate nuclei were intact, and 65 in case of bilateral damage of the dentate nuclei. The deleterious effect of radiotherapy on brain development is well known, but in this study adjuvant treatments were comparable for these groups and IQ score was related to the presence of lesions of the cerebellar nuclei and the severity of the motor score. One possible explanation for the IQ reduction from deep nuclear lesions is that cognitive impairment occurs as a consequence of motor disruption. However, the correlation of cognitive and motor deficits may also be due to the presence of both cognitive and motor "channels" [84] in close proximity to each other within the relatively confined region of the dentate nucleus.

Cerebellar dysfunction may be more deleterious during early brain development. In fact, there are two disorders with prominent cerebellar lesions which present with reduction in general intelligence: children and adolescents with congenital cerebellar disorders and children born very preterm. In children with congenital malformation of the cerebellum significantly reduced IQ has been found ([80,82] for review). Tavano et al. [86] performed neuropsychological tests in 27 children, adolescents and young adults with congenital cerebellar malformations. Wechsler Intelligence test scores for the appropriate age range were used. 74% of the patients presented with mental retardation (IQ $<$ 70). Five patients showed borderline IQ and only two had a normal IQ. Findings were most marked in the group of patients with vermal agenesis and diffuse cerebellar hypoplasia, and less in patients with agenesis or dysplasia of one or both cerebellar hemispheres. This is unexpected given the general notion that the posterior cerebellar hemispheres, but not the vermis contribute to cognition [83]. Likewise it is surprising that patients with almost complete absence of the cerebellum perform in general better than patients with lesions restricted to the vermis. Tavano et al. [86] reported one case with cerebellar agenesis. His cognitive level was just below the limits of norm (IQ 69). Findings agree with IQ measures based on Wechsler scale in another single case of cerebellar agenesis (IQ 66 [65]). In the latter case, based on Raven's SPM test IQ was 87 [90].

However, motor development and performance is impaired in these patients, and some of them show slow improvement. It is difficult to know if impaired cognitive development and neuropsychological test performance were directly related to lack of cerebellar function, or caused by motor disorders. Another challenge is the exclusion of extracerebellar lesions. For example in a group of 34 patients with non-progressive congenital cerebellar disorders Steinlin et al. [79] reported spasticity, focal dystonia and epilepsy in a significant number of patients. These symptoms could indicate involvement of extracerebellar areas. Alternatively, absence of normal cerebellar input to neocortical areas could contribute to IQ reduction. The influence of cerebellum on neocortical function should not be discounted when assessing the role of the cerebellum in cognitive function. For example, abnormalities of white matter development in cerebral areas that have been reported in a subset of patients may reflect consequences of altered cerebellar input.

Similar challenges apply to the interpretation of findings in very preterm children with cerebellar disease. Mental retardation in very preterm children has been related to bilateral extensive white matter reduction, or in a small number of cases, to cerebellar atrophy. Krägeloh-Mann et al. [45] reported three cases with marked mental retardation and cerebellar atrophy. The authors state that these cases were not accompanied by clincial cerebellar signs. The conclusion was made that very early acquired cerebellar lesions may be compensated with regard to motor signs, whereas mental dysfunctions may be sustained. However, two of the three patients revealed microcephaly and decreased brain growth may be related to mental retardation [45,46]. Furthermore, in the three cases white matter changes were also present.

In the subsequent years cerebellar injury in the preterm infant became increasingly recognized. These lesions have a prominent hemorrhagic component and have been termed cerebellar hemorrhagic injury [49]. Very preterm children with extremely low birth weight (that is infants born before 32 weeks of gestation and weighing $<$ 750 g) are at highest risk with an incidence as high as 19%. Isolated cerebellar hemorrhage without obvious associated supratentorial parenchymal lesions are not uncommon. These lesions lead to persistent cerebellar injury. As a result, in later MRIs destruction of the cerebellum develops to various degrees. Children have been investigated at a maximum age of 36 months. No formal IQ testing has been done, but significant deficits in cognition have been reported in these children [49]. Although, as stated by the authors, it is possible that more subtle forms of supratentorial parenchymal injury (particulary in the cerebral white matter) may have gone undetected, such results are consistent with the hypothesis that very early cerebellar lesions can result in significant reduction of cognitive development.

Mild IQ differences (IQ is on average 10 points below full-term children) are also observed in preterm children without focal brain lesions [46]. Abnormal development of the cerebellum may contribute. Allin and coworkers report a reduced cerebellar volume in preterm adolescents [57]. They found a positive correlation between cerebellar volume and IQ measures. Similar to Krägeloh-Mann et al. [45] no obvious cerebellar motor signs were reported. Shah et al. [75], on the other hand, found that cerebellar volumes were not different in preterm infants from term infants if cerebellar volumes were related to total intracranial volume. In that study neurodevelopment was principally related to white matter injury.

In summary, cerebellar damage or dysfunction has produced considerably variable effects on general intelligence. Relatively few people with cerebellar disease acquired after birth show a general decline in intelligence, with most studies reporting performance within the normal range and the majority of studies reporting no significant differences comparing patients and controls. Significant disorders have been reported in some patients with focal lesions, but not in others; for the latter group a possible explanation is that critical cognitive regions of the posterior cerebellar hemispheres [83] have been spared. A decline of IQ has been reported in acute onset cerebellar disease, but tends to be transient, and cerebro-cerebellar diaschisis may play a role [55]. More marked impairment of general intelligence has been found in patients with congenital disorders and in very preterm children with acquired cerebellar lesions. Although the consequences of abnormal motor abilities during development as well as potential presence of extracerebellar damage on cognition in these patients must receive further study, it is possible that very early cerebellar lesions can have a marked impact on cognitive development.

## 4. Verbal working memory

There is very good reason to believe that the cerebellum is involved in specific cognitive tasks. A good example is verbal working memory. Neuroanatomical, human cerebellar lesion and neuroimaging studies provide good evidence that the cerebellum contributes to verbal working memory, which in turn may influence performance in a range of other cognitive domains ([10, 11] for recent reviews, [78]). However, many patients perform within the normal range on standard tests and decline on a group level is generally mild and not always significant when cerebellar subjects and healthy controls are compared (Table 2).

In standard neuropsychological assessment, Wechsler Memory Scale (WMS) forward and backward digit span test is commonly applied to assess the function of the verbal working memory [99]. Digits of increasing length are read aloud with a frequency of 1 per second. Participants are asked to reproduce the digits immediately thereafter in either a forward or backward manner. In the WMS-revised subtest, the starting length is three items for the forward digit span and two items for the backward digit span. Two trials of a given digit length are performed. Digits are different between each trial. If the participant recalls all of the items of at least one of the two trials in the correct order, digit length is increased by one item. Testing ends when participants make an error on both trials of a given length. Both digit span forward and backward include a maximum of twelve trials. Results can be given as scores (scoring 1 for each correct trial, that is maximum score is 12 for digit span forward and backward, total maximum score is 24) or span length (largest number of items a participant is able to reproduce accurately, that is eight in forward span, seven in backward span, total maximum 15).

In the human cerebellar lesion literature, the results are inconsistent. There are a number of studies, which did not observe disorders in digit span in cerebellar patients. Our own group tested digit span in patients with degenerative cerebellar disorders. In none of these studies a significant difference between cerebellar subjects and IQ matched controls were observed [63,89, 91]. Group sizes were comparatively small (8–12). Because lesions are chronic, effects of compensation may have occurred. However, similar negative findings have been reported in a larger group of patients with SCA6 ($n$ = 18; [85]). Furthermore, preserved digit span has also been reported in subjects with acute ischemic lesions [38,51].

Mild abnormalities have been reported by others. Maddox et al. [50] described in a group of patients with degeneration and focal lesion digit span below controls. Group difference approached significance ($p = 0.053$). Ziemus et al. [103] examined nine patients with cerebellar infarct and reported abnormal digit span in five of the patients.

Overall, abnormalities appear to be more pronounced in children with surgical lesions of the cerebellum, and the same considerations described above regarding the potential presence of hydrocephalus also apply in these cases. Scott et al. [74] reported significant abnormalities in children with cerebellar tumors. Group size, however, was small and a subset of children had received adjuvant chemo- and radiotherapy because of malign tumors. Vaquero et al. [97] compared digit span in children with chronic surgical lesions for benign astrocytomas and malign medulloblastomas. Medulloblastoma patients were more severely affected, but astrocytoma patients showed also deficits in digit span. Both groups performed significantly below controls. Steinlin et al. [81] examined 24 children after removal of benign cerebellar tumors. They report significantly reduced group mean values compared to normative data with the majority of children performing within the normal range.

In all studies cited above, forward and backward digit span were combined. Disorders may be more marked in the more difficult backward compared to the forward span. Digits span backward requires participants to maintain the information (direct order digits) while executing the secondary task (inversing the digits order). Again, findings are inconsistent. We examined 16 children and adolescents with chronic surgical lesions for benign cerebellar tumors. Forward and backward digit spans were not significantly different from controls [44]. Justus et al. [41] reported forward and backward digit span within the normal range in nine cerebellar patients both with focal and degenerative cerebellar lesions. Leggio et al. [48] analyzed forward and backward digit span separately in two large groups of chronic cerebellar patients ($n = 60$ and $n = 34$). Patients' performance is reported to be within the normal range. The authors, however, do not report statistical comparisons between cerebellar and control groups.

Globas et al. [31] examined 12 patients with SCA6. Group mean backward digit span, but not forward span was below the controls. The difference did not reach significance. Exner et al. [26] examined eleven patients with cerebellar stroke. Patients with lesions within the PICA but not the SCA territory performed on average worse than the controls in the backward tasks. Again, this difference did not reach significance. The same trend was reported by Ben-Yehuda and Fiez [12] in a small group of six patients with focal lesions: Although there was no significant group difference there was a trend towards a smaller mean span in the patient group for the digit-backward condition ($p = 0.08$). Lack of statistical power in these small groups likely contributed to trends not reaching statistical significance.

The latter group [61] examined 15 patients with focal cerebellar lesions due to stroke or tumor resection. They found a significant deficit in verbal working memory. On a group level, both forward and backward verbal spans were lower than in controls. Disorders tended to be more marked in backward verbal spans. Importantly, all cerebellar patients performed within the normal range. Witt et al. [101] reported unimpaired forward digit span in 16 patients with cerebellar degeneration, but significantly reduced backward digit span compared to controls. The finding of preserved forward but impaired backward digit span has also been reported in a case of paraneoplastic cerebellar degeneration [17]. Silveri et al. [77] observed reduced forward and backward digit span in a patient two weeks after a right- hemisphere cerebellar tumor resection, but within the normal range five months after surgery.

Differences between control and patient groups may be missed because large group sizes are needed to show subtle abnormalities, or cerebellar areas thought to be related to verbal working memory may have been spared. Vaquero et al. [97] reported that findings were more severe in children where lesion included the dentate nucleus. This fits to the observation that motor disorders are more marked if the corresponding cerebellar nuclei are affected [44,73]. Ravizza et al. [61] showed a significant negative correlation between damage of the inferior cerebellum and disorders in verbal digit span. Because cerebellar lesions did not reduce behavioural markers of rehearsal (e.g. articulatory suppression, word length), the authors concluded that phonological encoding, but not articulatory rehearsal appeared to be the problem in their group of cerebellar patients.

In accordance with the Ravizza study, Kirschen et al. [43] found that reduced verbal digit span was correlated with damage to the hemispheral lobule VIII (which is part of the inferior cerebellum) in twelve children with tumor surgery. In this study, however, patients also showed reduced effects of articulatory suppression and this was correlated with damage to the vermis and hemispheral lobules IV/V bilaterally (which is part of the superior cerebellum).

Table 2

Summary of digit span reported in human cerebellar lesion studies. [Wechsler Memory Scale – Revised [98]: raw score: maximum digit span (max. forward 8, max. backward 7, sum 15); standard score: scoring 1 for each correct trial (max. forward 12, max. backward 12, sum 24); scaled score: transformation in scaled scores (normal mean 10 SD 3)]

| Study | Cerebellar condition (n/age) | Digit span | Group comparison |
|---|---|---|---|
| *Adults: cerebellar degeneration* | | | |
| Timmann et al. [89] | - SAOA, ADCAIII, SCA6 (n = 9; 56.67 SD 9.41 yrs.)<br>- controls (n = 9; 55.0 SD 8.46 yrs.) | raw score forward:<br>- cerebellar: 5.89 SD 1.27<br>- controls: 6.22 SD 0.83 | p = 0.52 (t test) |
| Witt et al. [101] | - cerebellar degeneration (n = 9 pure cerebellar degeneration, n = 4 SCA1 or SCA2, n = 3 OPCA; 53.38 SD 14.71 yrs.)<br>- controls (n =20; 59.81 SD 7.58 yrs.) | raw score forward:<br>- cerebellar: 5.63 SD 1.15<br>- controls: 7.33 SD 1.02<br>raw score backward:<br>- cerebellar: 3.63 SD 0.96<br>- controls: 5.57 SD 0.98 | forward n.s.<br>backward < 0.05 (t test) |
| Globas et al. [31] | - SCA6 (n = 12; 57.5 SD 10.4 yrs.)<br>- controls (n = 12; 59.0 SD 5.6 yrs.) | raw score forward:<br>- SCA6: 6.6 SD 1.0<br>- controls: 6.9 SD 1.3<br>raw score backward:<br>- SCA6 4.8 SD 1.5<br>- controls 5.6 SD 1.4 | forward: p = 0.49<br>backward p = 0.22<br>(oneway-ANOVA) |
| Maschke et al. [56]<br>Timmann et al. [91] | - SAOA, ADCAIII, SCA6, EA2 (n = 8; 52.2 SD 6.2 yrs.)<br>- controls (n = 8; 52.9 SD 7.9 yrs.) | raw score sum forward and backward:<br>- cerebellar: 12.00 SD 3.24<br>- controls: 13.38 SD 3.29 | p = 0.899 ( t test) |
| Richter et al. [63] | - SAOA, ADCAIII, SCA6, EA2 (n = 12; 50.3 SD 10.4 yrs.)<br>- controls (n =12; 53.3 SD 10.9 yrs.) | standard score sum forward and backward:<br>- cerebellar: 15.2 SD 3.5<br>- controls: 16.1 SD 2.9 | p > 0.15 (t test) |
| Maddox et al. [50] | - cerebellar degeneration (n = 8) and stroke (n = 6) (45-79 yrs.)<br>- controls (n = 14; 58-75 yrs.) | scaled scores of raw scores:<br>- cerebellar: 9 SD 3<br>- controls: 11.3 SD 2.3 | p = 0.053 (t test) |
| Suenaga et al. [85] | - SCA6 (n = 18; 64.1 SD 11.9 yrs.) | raw score sum forward and backward: | n.s. (Mann-Whitney U |

Table 2, continued

| | | | |
|---|---|---|---|
| | - controls (n = 21; 63.0 SD 9.4 yrs.) | - cerebellar: 12.5 SD 2.8<br>- controls: 13.2 SD 4.0 | test) |
| *Adults: chronic focal lesions (mainly cerebellar stroke)* | | | |
| Exner et al. [26] | - PICA (n = 6; 62 SD 5 yrs.)<br>- SCA (n = 5, 55 SD 3 yrs.)<br>- controls (n = 11; 57 SD 5 yrs.) | standard score forward:<br>- PICA: 7 SD 2<br>- SCA: 9 SD 1<br>- controls: 7 SD 1<br>standard score backward:<br>- PICA: 5 SD 2<br>- SCA: 7 SD 2<br>- controls: 6 SD 1 | n.s. (Kruskal Wallis Test) |
| Justus et al. [41] | - focal lesions (n = 6) and cerebellar degeneration (n = 4) (age 44-78)<br>- controls (n = 10; mean age 67 years) | raw score forward:<br>- cerebellar: 5.25 SD 1.6<br>raw score backward:<br>- cerebellar: 3.3 SD 1 | - low normal range: 5/10;<br>- normal range: 4/10;<br>- missing data: 1/10 |
| Ravizza et al. [61] | - focal lesions (stroke: n = 10; tumor/cyst resection: n = 5; 60.3 SD 14.6 yrs.)<br>- n = 15 controls (n = 15; 61.2 SD 15.2 yrs.) | raw score forward (Fig. 3 in [60]):<br>- cerebellar: mean ca. 6<br>- controls: mean ca. 7<br>raw score backward (Fig. 3 in [60]):<br>- cerebellar: mean ca. 4<br>- controls: mean ca. 5.5 | in the normal range but:<br>- 3-way interaction (verbal vs. spatial x forward vs. backward x cerebellar vs. contols): p < 0.05<br>- con. vs. cer. p < 0.005 (t test) |
| Ziemus et al. [103] | - cerebellar stroke (n = 9; 46.2 SD 8.1 yrs.) | Z scores forward and backward:<br>- n = 5: Z score > 1<br>- n = 4: Z score <= 1 | Z score difference greater than –1 was considered abnormal |
| Leggio et al. [48] | - right focal lesions (n = 21; 53.29 SD 18.44 yrs.)<br>- left focal lesions (n = 21; 49.76 SD 17.98 yrs.)<br>- cerebellar degeneration (n = 18; 42.88 SD 12.81)<br>- controls (n = 69; 43.78 SD 15.96 yrs.) | raw score forward:<br>- right cerebellar 5.52 SD 0.93<br>- left cerebellar 6.15 SD 0.81<br>- degeneration 5.61 SD 1.20<br>- controls 5.76 SD 1.01<br>raw score backward:<br>- right cerebellar 4.00 SD 1.22 | cerebellar groups performed above cut-off:<br>forward: cut-off = 5<br>backward cut-off = 3 |

Table 2, continued

| | | | |
|---|---|---|---|
| | | - left cerebellar 4.10 SD 1.02<br>- degeneration 3.67 SD 1.03<br>- controls 3.93 SD 1.10 | |
| Leggio et al. [48] | - right focal lesions (n = 11; 48.18 SD 20.72 yrs.)<br>- left focal lesions (n = 9; 60.63 SD 7.42 yrs.)<br>- cerebellar degeneration (n = 14; 49.36 SD 11.08 yrs.)<br>- controls (n = 132; 47.02 SD 17.33 yrs.) | raw score forward:<br>- right cerebellar 5.82 SD 0.60<br>- left cerebellar 5.78 SD 0.67<br>- degeneration 5.29 SD 1.59<br>- controls 5.59 SD 1.13<br>raw score backward:<br>- right cerebellar 4.36 SD 1.12<br>- left cerebellar 4.11 SD 1.17<br>- degeneration 3.43 SD 1.60<br>- controls 3.91 SD 1.38 | cerebellar groups performed above cut-off:<br>forward: cut-off = 5<br>backward cut-off = 3 |
| Ben-Yehuda and Fiez [12] | - focal lesions (n = 6; mean age 63.5 yrs.)<br>- controls (n = 6; mean age 59.7 yrs.) | raw score forward:<br>- cerebellar: 6.2 SD 1.0<br>- controls: 6.7 SD 0.8<br>raw score backward:<br>- cerebellar: 3.8 SD 0.8<br>- controls: 5.0 SD 1.3 | forward: n.s.<br>backward: p = 0.081<br>(t test) |
| *Adults: acute focal lesions* | | | |
| Schmahmann and Sherman [70] | n = 20 (48.2 range 23-74 yrs.):<br>- acute stroke (n = 12)<br>- acute tumor removal (n =1)<br>- chronic stroke (n = 1)<br>- acute cerebellitis (n = 3)<br>- cerebellar degeneration (n = 3) | Z score<br>-0.51 (SD 1.3) | compared to normative data:<br>p = 0.1501 |
| Silveri et al. [78] | - medulloblastoma right cerebellar hemisphere (n = 1; 18 yrs.) | - before surgery<br>raw score forward: 4; backward: 3<br>- 3 days after surgery<br>raw score forward: 4; backward: 3<br>- 5 months after surgery<br>raw score forward: 7; backward: 7 | below cut-off before and 3 days after surgery<br>(cut-off forward: 7 SD 2;<br>cut-off backward: 5 SD 2) |

Table 2, continued

| | | | |
|---|---|---|---|
| Hokkanen et al. [38] | - acute cerebellar stroke (n = 26; 47 SD 11 yrs.)<br>- controls (n = 14; 46 SD 11 yrs.) | age-adjusted standard scores forward and backward:<br>acute assessment<br>- cerebellar: 12.46 SD 2.85<br>- controls: 11.50 SD 3.01<br>3 months<br>- cerebellar: 13.5 SD 3.08<br>- controls: 11.71 SD 2.95 | effect of time $p < 0.05$ group effects n.s. (MANOVA) |
| Collinson et al. [17] | - paraneoplastic cerebellar degeneration (n =1; 63 yrs.) | raw/max. score forward: 8/16<br>raw/max. score backward: 2/14 | forward within standard norms, backward below standard norm of 6 |
| *Children: chronic focal lesions (surgery for benign cerebellar tumors)* | | | |
| Steinlin et al. [81] | - benign cerebellar tumors (n = 24; mean 15.04 yrs.) | scaled score forward and backward:<br>- cerebellar: 8.8 | compared with normative data: $p < 0.05$ ( t test) |
| Kirschen et al. [43] | - astrocytoma (n = 12; 12.5 SD 4.1 yrs.)<br>- controls (n = 12; age-matched) | raw score forward (Fig. 3 in [42]):<br>- cerebellar: 5.2<br>- controls: 5.9 | $p = 0.023$ (t test) |
| *Children: chronic focal lesions (surgery for benign vs. malign cerebellar tumors)* | | | |
| Scott et al. [74] | - cerebellar tumor surgery (n = 7; all but one had radiotherapy, none chemotherapy; mean age 9 yrs. 1 month) | Z scores (digit span done in 6/7)<br>- n = 3: Z scores = -1<br>- n = 3: Z scores = -1.3 | Z score difference greater than –1 was considered abnormal |
| Konczak et al. [44] | - cerebellar tumor surgery, (n = 14 benign tumors; n = 8 malign tumors with adjuvant chemo- and radiotherapy; mean age 10.28 yrs.)<br>- controls (n =14; 11-28 yrs.) | standard scores forward:<br>- benign: 6.5 SD 1.9<br>- malign: 6.7 SD 1.5<br>- controls: 7.7 SD 1.9<br>standard scores backward:<br>- benign 5.7 SD 1.3<br>- malign 5.2 SD 1.7<br>- controls 6.1 SD 1.4 | $p > 0.05$ |
| Vaquero et al. [97] | - astrocytoma (n = 29; 17.43 SD 7.11 yrs.)<br>- medulloblastoma (n = 29; radio- and chemotheray; 23.54 SD 8.33 yrs.) | standard score sum forward and backward:<br>- astrocytoma: 9.23 SD 3.08<br>- medulloblastoma: 8.57 SD 4.72<br>- controls: 15.24 SD 2.63 | controls vs. astrocytoma: $p = 0.003$; con. vs. medulloblastoma: $p = 0.017$ (Mann-Whitney U) |

ADCA III autosomal dominant cerebellar ataxia type III („pure cerebellar“); EA2 episodic ataxia type 2; OPCA olivopontocerebellar atrophy; PICA stroke within the territory of the posterior inferior cerebellar artery; PIQ performance intelligence quotient; SAOA sporadic adult onset ataxia; SCA stroke within the territory of the superior cerebellar artery; SCA1,2 spinocerebellar ataxia type 1,2 (extracerebellar involvement is common); SCA6 spinocerebellar ataxia type 6 („pure“ cerebellar); SD standard deviation.

These findings are in good accordance with neuroimaging data of Desmond and coworkers that the inferior cerebellum (together with the inferior parietal cortex) is involved in phonological encoding, whereas the superior cerebellum (together with the frontal lobe, that is Broca's area, left premotor area, supplementary motor area) contributes to subvocal rehearsal mechanisms of verbal working memory [16]. The latter agrees with findings by Ackermann and coworkers that the cerebellum is involved in the temporal organization of a prearticulatory verbal code ('inner speech') [3].

In summary, similar to findings of IQ measures, verbal working memory disorders appear to be somewhat more marked in children with cerebellar tumor removal. Adult cerebellar patients frequently perform within the normal range in standard verbal working memory tasks, in contrast to patients with damage to relevant cerebral regions (such as lesions in the extended Broca's area in frontal lobe [96]). However, significant verbal working memory impairments in adult cerebellar patients relative to controls have been observed.

## 5. General discussion

This review suggests that cognitive impairments in patients with cerebellar disease or damage can vary from significant to subtle, may be task dependent, and may possibly be specific to etiology. The subtle effects that are often observed in patient studies are at variance with anatomical and functional brain imaging data, which provide good evidence that the cerebellum contributes to cognitive function [84]. There are at least three possible reasons for the discrepancy. Firstly, in the majority of studies patients with chronic disease have been included. More marked and often transient cognitive dysfunctions have been reported in individual patients with acute cerebellar disease [17,53,54,66,70, 76]. Findings in acute disorders could be influenced by cerebro-cerebellar diaschisis, that is an abrupt disconnection syndrome, where disorders represent dysfunction of the connected cerebral area [55]. Alternatively, more marked effects from acute disorders could also be due to the fact that there has been less time for other brain systems to compensate for cerebellar damage, in contrast to chronic cerebellar disease. Compensation mechanisms could also explain the frequently transient nature of cognitive (as well as motor) dysfunction arising from cerebellar damage.

A second reason for the discrepancy may be the topographic organization of different motor and nonmotor function in cerebellum [83]. Focal cerebellar lesions which spare the areas within the posterior lobe known to be critical for nonmotor function are unlikely to present with significant cognitive signs. A similar explanation may account for some of the inconsistent findings in diffuse cerebellar degeneration. That is, degenerative disorders affect primarily the vermis and anterior lobe, and to a lesser extend the posterior hemispheres [15]. This may be one reason why motor disorders in patients with degenerative disease are marked, whereas cognitive function is largely preserved.

Finally, the cerebellum may be more important during early cognitive development than cognitive performance in adulthood. In fact, cognitive dysfunction appears to be most marked in children with congenital cerebellar disease, and more marked in children than in adults with acquired cerebellar disease. In congenital disease cerebellar motor dysfunctions are largely missing. The developing brain may be better equipped to compensate for lesions of primary motor than cognitive areas. Although the contribution of extracerebellar lesions as well as the influence of impaired motor function on cognitive function during development must be carefully assessed, the relatively profound effects on cognition of cerebellar damage during early development may offer important clues as to the nature of cerebellar cognitive functions. Future studies are therefore needed to explore the developmental influences of the cerebellum on cognitive function.
